# Prevalence and burden of obstructive lung disease in the urban poor population of Ottawa, Canada: a community-based mixed-method, observational study

**DOI:** 10.1186/s12889-021-10209-w

**Published:** 2021-01-21

**Authors:** Smita Pakhale, Saania Tariq, Nina Huynh, Sadia Jama, Tina Kaur, Catherine Charron, Kelly Florence, Fozia Nur, Margaret ( Ella) Bustamante-Bawagan, Ted Bignell, Robert Boyd, Joanne Haddad, Tetyana Kendzerska, Gonzalo Alvarez

**Affiliations:** 1 Department of Medicine, The Ottawa Hospital, 501 Smyth Road, Ottawa, Ontario K1H 8L6 Canada; 2 The Ottawa Hospital Research Institute (OHRI), Ottawa, Canada; 3 University of Ottawa, Ottawa, Canada; 4 Community (peer) Researcher, The Bridge Engagement Centre, Ottawa, Canada; 5 Oasis, Sandy Hill Community Health Centre, Ottawa, Canada; 6 Canadian Mental Health Association, The Ottawa Branch, Ottawa, Canada

**Keywords:** Urban poor, Social determinants of health, Obstructive lung disease

## Abstract

**Background:**

Globally the burden of Obstructive Lung Diseases (OLD) is growing, however its effect on urban poor populations with the high prevalence of tobacco dependence is virtually unknown. The purpose of this project is to estimate the prevalence and burden of OLD in the urban, low-income populations of Ottawa, Canada.

**Methods:**

The study presented in this paper was part of the PROMPT (Management and Point-of-Care for Tobacco Dependence) project; a prospective cohort study in a community-based setting (*n* = 80) with meaningful *Patient Engagement* from design to dissemination. Spirometry data, standardized questionnaires and semi-structured interviews from PROMPT were interpreted to understand the lung function, disease burden and social determinants (respectively) in this population.

**Results:**

The prevalence of OLD among those who completed spirometry (*N* = 64) was 45–59%. Generic and disease-specific quality of life was generally poor in all PROMPT participants, even those without OLD, highlighting the higher disease burden this vulnerable population faces. Quality of life was impacted by two major themes, including i) socioeconomic status and stress and ii) social networks and related experiences of trauma.

**Conclusion:**

The prevalence and disease burden of OLD is significantly higher in Ottawa’s urban poor population than what is observed in the general Canadian population who smoke, suggesting an etiological role of the social determinants of health. This urges the need for comprehensive care programs addressing up-stream factors leading to OLDs, including poor access and utilization of preventive healthcare addressing the social determinants of health.

**Trial registration:**

ClinicalTrails.gov - NCT03626064, Retrospective registered: August 2018.

**Supplementary Information:**

The online version contains supplementary material available at 10.1186/s12889-021-10209-w.

## Background

In the past decade, researchers have documented a growing socioeconomic inequity leading to poverty and homelessness among a group of people living in urban settings in wealthy countries (the urban poor) [1]. This characteristic low socioeconomic status (SES) in this population has been associated with co-addiction of licit and/or illicit drugs, including tobacco [2–4]. This includes tobacco cigarettes, which have led to a high prevalence of nicotine dependence and other tobacco-related diseases in this population [5, 6]. Previous studies working with these vulnerable groups have found that 70–96% of their cohort’s smoke tobacco [6–9]. A greater mortality with a higher population attributable fraction for smoking tobacco is also seen in those with low SES compared to those with high SES [4, 10]. Due to the higher rates of tobacco and substance use, chronic Obstructive Lung Diseases (OLDs), such as Chronic Obstructive Pulmonary Disease (COPD), asthma, and bronchiectasis, are expected to be a growing problem in the urban poor [3, 4, 11].

While many OLDs are preventable and treatable, there is a problematic knowledge gap of undiagnosed OLDs [12, 13]. OLDs impose a significant burden on individuals through progressive symptoms, acute pulmonary exacerbations, worsening quality of life, and premature death [12]. Social disparities such as education, occupation, racialization, and healthcare access are known to contribute to the differences in OLD-related health status in minority groups across the United States [14, 15]. However, there is minimal research that investigates the prevalence of OLDs in low-income, vulnerable populations. To our knowledge, only one small cohort study conducted in a San Francisco homeless shelter revealed that 15% of the 68 adults had spirometry-diagnosed OLD [5]. Furthermore, there is a poor understanding of the social determinants of health (SDH) and OLD disease burden in these vulnerable groups. Hence, this paper primarily aims to estimate the prevalence and disease burden of OLDs, mainly asthma and COPD, in Ottawa’s urban poor population using pre- and post-bronchodilator spirometry and patient-reported health measures. Secondly, we aim to explore the SDH and experience of people living with OLD in this urban poor population to further elucidate the quality of life burden of OLDs.

## Methods

Data was obtained from the Participatory Research in Ottawa: Management and Point-of-Care for Tobacco Dependence (PROMPT) Project, a prospective cohort study. PROMPT’s target population included people experiencing homelessness, at-risk for homelessness (i.e., insecurely housed due to low or insecure income, and/or mental health issues, or legal challenges) or using poly-substances. The PROMPT inclusion criteria were: 1) currently living in Ottawa, Canada for at least 3 months prior to enrolment, 2) 16 years or older, 3) have used street drugs in the past year (other than recreational marijuana or alcohol), and 4) have smoked tobacco in the past 7 days. Exclusion criteria included: 1) declined consent 2) current use of in-person addictions treatment (and therefore unavailable for follow-up), 3) current or recent (past 30 days) enrollment in a smoking cessation program and 4) terminal illness, with a life expectancy of < 3 months. During a 6-month treatment period, follow-up data, including patient-reported substance use and other health measures were collected. During PROMPT, pre- and post-bronchodilation spirometry was performed at baseline; analysis of which is presented here. Additionally, post-study semi-structured interviews were conducted with a subset of participants (recruited through convenience sampling) with study-spirometry diagnosed OLD. The project was approved by the Ottawa Health Science Network Research Ethics Board and written informed consent was obtained from all participants.

### Patient and community engagement

The PROMPT study occurred in partnership with community (peer) researchers; people with lived experience truly representative of the target population (e.g., people who smoke, are homeless or at-risk for homelessness or use poly-substances). Peers underwent intensive training, including study protocol (consent, recruitment, and research ethics), conducting semi-structure interviews and administrating spirometry. Peers used a social-network approach to recruit participants and completed baseline enrollment, including administering informed consent, an iPad-based questionnaire, and hand-held spirometry.

All participants had access to free nicotine replacement therapy, one-on-one nurse counselling twice a week, ongoing peer support, peer-led weekly life-skills workshops, and access to a safe, non-judgmental, low-threshold community-based research space (“*The Bridge”* Engagement Centre) for 6 months. Peers administered monthly follow-up surveys, spirometry at the last follow-up, and a post-study semi-structed interview. Additional details on the design to dissemination *Patient Engagement* model, the ‘Ottawa Citizen Engagement and Action Model’ (OCEAM) [16] and PROMPT project details, are published elsewhere [7, 17].

### Data and measures

Data on seven patient-reported measures, administered by the peers to all participants at baseline included: 1) demographic questionnaires designed with peers (including detailed smoking history, drug use, and social network) (see Additional File 3*)*, 2) BOLD core questionnaire used in the CanCOLD study, 3) COPD Assessment Test (CAT), 4) EQ-5D-3L to measure quality of life, 5) Patient Health Questionnaire (PHQ-8), and 6) the Generalized Anxiety Disorder (GAD-7) questionnaire (see Additional File 2). Participants who consented and were physically able underwent pre- and post-bronchodilator (before and after inhaling 200 μg of salbutamol) spirometry to assess lung function. Prior to spirometry, participants completed spirometry safety questions to rule out any contraindications [18]. Peers conducted all hand-held spirometry at the Bridge, after receiving a standardized hand-held spirometry training from the study respirologist (SP). Peers were required to achieve at least 8 grade A or B maneuvers (programmed by the ‘EasyOne’ hand-held diagnostic spirometer from NDD Medical Technologies) to maintain test quality and received regular feedback from the study respirologist, who was onsite 4 times per week alongside the study nurse and study coordinator. Further details on the spirometry training and administration have been previously published [17]. Lastly, we designed a post-study semi-structure interview guide in partnership with the peers, consisting of a series of open-ended questions regarding health, income, education, health services, and the surrounding physical environment. The use of open-ended questions allowed participants to freely share their feelings and attitudes about different aspects of their lived experience that may be relevant to the topics at hand.

### Interpretation of Spirometry

To estimate predicted values of spirometry (adjusting for age, sex, height and weight) we utilized The National Health and Nutrition Examination Survey (NHANES) III formula [19]. Spirometry interpretation was according to the American Thoracic Society/European Respiratory Society (ATS/ERS) interpretative strategies [18]. Two spirometry interpretation methods labeled participants with COPD: fixed ratio (a post-bronchodilator Forced Expiratory Volume in 1 s/ Forced Vital Capacity (FEV_1_/FVC) ratio < .70) and the Lower Limit of Normal (LLN) (a post-bronchodilator FEV_1_/FVC ratio ≤ LLN). To calculate the LLN, we used the lower 5th percentile of FEV_1_/FVC ratio using NHANES III reference equations [19]. Due to lack of appropriate reference data based on race, including Indigenous and East Asian origin, Caucasian reference values were only used. Participants labeled with Asthma showed significant reversibility, defined as pre-bronchodilation FEV_1_/FVC ratio < .70 or ≤ LLN with an improvement of ≥12% and 200 cc in FEV_1_ or FVC post-bronchodilation.

### Statistical analysis

We completed descriptive statistics to estimate baseline population characteristics. To calculate questionnaire specific score and proportions we used the scoring methods of the CAT, EQ-5D-3L, PHQ-8, and GAD-7. Lastly, we calculated confidence intervals (CI) at 95% for disease prevalence and burden.

### Qualitative analysis

Anonymized interview transcripts were analysed using inductive thematic analysis with NVivo Software 12. Codes generated by two independent coders were compared and a final list of codes and overarching themes was determined. Resulting themes were used to gain a better understanding of the population characteristics and further explain quantitative results.

## Results

From March to August 2016, 2 to 4 peers recruited 80 participants from the Ottawa urban poor population, with less than 5% of potential participants contacted but not enrolled. Of the 80 participants, 67 completed spirometry at baseline, while 13 participants did not complete spirometry because of contraindications. Additionally, we removed 3 test results from the data set because of poor-quality spirometry tests (i.e., over 40% decrease in post-bronchodilation performance or inconsistent FEV_1_ and FVC outputs). Therefore, the analysis was conducted with 64 participants with complete spirometry data. Lastly, post-study interviews were completed with 11 participants who had study spirometry diagnosed OLD.

There were no significant differences in the baseline characteristics of the whole cohort compared to those who underwent spirometry (Table 1).

**Table 1:** Demographic characteristics of the PROMPT cohort participants

Characteristic	Participants with spirometry (*N* = 64)	All participants (*N* = 80)
**Sex** (male)	69%	70%
**Ethnicity**		
Caucasian	78%	78%
First Nations	16%	16%
Inuit	1.5%	1%
Métis^b^	1.5%	1%
East Asian	1%	1%
Other	2%	3%
**First language**		
English	90%	83%
French	9%	12%
Other	1%	4%
N/A^a^	0%	1%
**Age**		
16–30	18%	15%
31–40	9%	9%
41–50	42%	44%
51–65	31%	31%
N/A^a^	0%	1%
**Education**		
Elementary/high school	36%	34%
High school graduate/GED	28%	31%
Some college or university	27%	26%
College or university completed	6%	5%
None	1.5%	1%
N/A^a^	1.5%	3%
**Monthly Income**		
< $499	13%	11%
$500–$999	33%	34%
$1000–$1999	43%	44%
$2000–$2999	11%	10%
N/A^a^	0%	1%
**Food insecurity:**		
Always	18%	15%
Most of the time	10%	11%
Occasionally	15%	16%
Sometimes	25%	26%
Usually	12%	10%
Never	18%	19%
N/A^a^	2%	3%
**Number of cigarettes per day:**		
< 15	53.8%	32.5%
15–25	34.6%	42.5%
26–35	9%	10%
36–40	2.6%	6.3%
N/A^a^	0%	8.7%
**Total years tobacco smoking:**		
< 10	31.3%	12.5%
10–20	11.3%	15%
21–30	28.7%	33.7%
31–40	18.7%	25%
41–50	7.5%	7.5%
51–60	2.5%	2.5%
N/A^a^	0%	3.8%
**Previous or current drug use**		
Crack	72.7%	66.3%
Marijuana	60.6%	61.3%
Heroine	25.8%	23.8%
Fentanyl	13.6%	13.8%
Oxycontin	10.6%	12.5%

^a^N/A = missing or refuse to answer

^b^Métis - are a group of people in Canada who trace their descent to First Nations people and European settlers. They represent the majority of those identifying as Métis, though smaller communities also exist in the United States. They are recognized as one of Canada’s aboriginal or indigenous people under the Constitution Act of 1982, along with First Nations and Inuit people

### Disease prevalence

Approximately half of those with spirometry were considered to have OLD using the LLN (45, 95% CI: 30–60%) and the fixed ratio (59, 95% CI: 44–74%) diagnostic method. Of those spirometry results indicating OLDs, 14–16% indicated asthma (95% CI:7–25%; 95% CI: 5–23%) and 31–44% indicated COPD (95% CI: 20–42%; 95% CI: 33–55%), depending on spirometry diagnostic method. There was no significant difference between the two different spirometry diagnostic criteria (*p* > 0.05). Additionally, there was no significant difference in the pre-bronchodilator spirometry measurements (FEV_1_ and FVC) between those with OLD and those without OLD. However, there was a significant difference of FEV1/FVC ratios between those with OLD and those without OLD (*p* < 0.0001). As well, participants with significant bronchodilator response had a lower mean FEV_1_ in comparison to participants without an OLD (Table 2).

**Table 2:** Spirometry test results of the PROMPT cohort participants

Mean (SD)	Participants with spirometry (*N* = 64)	Participants without OLD (Fixed Ratio) on spirometry (*N* = 26)	Participants without OLD (LLN) on spirometry (*N* = 35)	Participants with OLD (Fixed Ratio) on spirometry (*N* = 38)	Participants with OLD (LLN) on spirometry (*N* = 29)	Participants with significant reversibility on spirometry (*N* = 10)
FEV_1_ (L)^a^	2.80 (1.04)	3.08 (0.90)	3.11 (0.97)	2.61 (1.11)	2.42 (1.02)	1.99 (0.80)
FEV_1_% Predicted	76.65 (23.64)	89.95 (19.49)	88.52 (19.41)	67.55 (22.04)	3.84 (0.73)	52.91 (17.48)
FVC^b^ (L)	4.15 (1.31)	3.91 (1.13)	4.08 (1.33)	4.32 (1.41)	4.25 (1.30)	3.67 (1.18)
FVC % Predicted	76.75 (23.64)	91.34 (20.96)	91.95 (21.21)	88.12 (17.88)	86.38 (16.01)	77.17 (18.03)
FEV_1_/FVC	0.68 (0.15)	0.79 (0.06)	0.77 (0.08)	0.60 (0.15)	0.57 (0.15)	0.54 (0.13)
FEV_1_ Pre- & Post-bronchodilator Difference (L)	−0.13 (0.71)	−0.13 (0.58)	−0.13 (0.56)	−0.13 (0.79)	−0.13 (0.87)	0.69 (0.35)
FEV_1_ Pre- &Post-bronchodilator Percent Difference (%)	−1.94 (23.29)	−3.49 (17.58)	−2.91 (19.15)	− 0.88 (26.69)	− 0.77 (27.80)	35.54 (12.99)

^a^*FEV*_*1*_ Forced Expiratory Volume in 1 s

^b^*FVC* Forced Vital Capacity

### Disease burden

Majority of participants reported symptoms of OLD as measured by the CanCOLD questionnaire, regardless of the presence of spirometry diagnosed OLD. This included cough (64%; 95% CI: 51–77%), phlegm (70%; 95% CI: 58–82%) shortness of breath (39%; 95% CI:27–51%) and, wheezing (71%; 95% CI: 59–83%). A greater percentage of participants with OLD and also those with significant reversibility had more frequently reported symptoms of cough, phlegm, and shortness of breath, than those without OLD. Similarly, all participants, regardless of OLD diagnosis, reported some respiratory symptoms (especially their cough) impacting their ability to function, as measured by the CAT score (Table 3).

**Table 3:** Self-reported and measured Obstructive Lung Disease and respiratory symptoms, related conditions of the PROMPT cohort participants

Questionnaire assessment	All Participants with spirometry (*N* = 64)	Participants without OLD (Fixed Ratio) on spirometry (*N* = 26)	Participants without OLD (LLN) on spirometry (*N* = 35)	Participants with OLD (Fixed Ratio) on spirometry (*N* = 38)	Participants with OLD (LLN) on spirometry (*N* = 29)	Participants with significant reversibility on spirometry (*N* = 10)
CAN-COLD ^a^ (percentage)						
Cough (with no cold)	64%	58%	60%	68%	69%	80%
**< 2 years** ^**b**^	8%	4%	6%	11%	10%	10%
**2–5 years** ^**b**^	13%	19%	17%	8%	7%	10%
**> 5 years** ^**b**^	31%	31%	11%	32%	28%	40%
Phlegm (with no cold)	70%	54%	60%	82%	83%	80%
**< 2 years** ^**c**^	17%	19%	20%	16%	14%	20%
**2–5 years** ^**c**^	9%	15%	11%	5%	7%	10%
**> 5 years** ^**c**^	20%	8%	11%	29%	31%	40%
Chest						
**Wheezing/Whistling**	72%	69%	74%	74%	69%	70%
**With cold**	33%	31%	29%	34%	38%	50%
Chest						
**Shortness of Breath**	39%	38%	37%	39%	41%	60%
**Unable to walk**	36%	38%	34%	34%	38%	40%
CAT (Mean/SD)						
Cough	4.03 (1.36)	4.00 (1.50)	4.09 (1.42)	4.05 (1.27)	3.97 (1.30)	4.20 (1.25)
Phlegm	3.92 (1.46)	3.85 (1.38)	4.06 (1.39)	3.97 (1.53)	3.76 (1.55)	4.20 (1.81)
Chest	3.06 (1.62)	2.88 (1.63)	3.00 (1.66)	3.18 (1.63)	3.14 (1.60)	3.40 (1.58)
Walk	3.34 (1.85)	3.35 (2.08)	3.34 (1.92)	3.34 (1.71)	3.34 (1.80)	3.80 (1.75)
Activities	2.55 (1.60)	2.54 (1.68)	2.63 (1.70)	2.55 (1.57)	2.45 (1.50)	3.20 (1.48)
Confident	2.47 (1.83)	2.85 (2.09)	2.54 (1.92)	2.21 (1.61)	2.38 (1.76)	2.00 (1.05)
Sleep	3.25 (1.88)	2.88 (1.99)	3.20 (1.97)	3.50 (1.78)	3.31 (1.79)	3.30 (1.83)
Energy	3.21 (1.61)	3.12 (1.61)	3.29 (1.56)	3.27 (1.63)	3.11 (1.69)	3.78 (1.79)
CAT score ^d^	25.78 (8.45)	25.46 (9.09)	26.14 (8.81)	26.00 (8.10)	25.34 (8.12)	27.5 (8.17)

^a^Canadian Chronic Obstructive Lung Diseases Study

^b^Duration (measured in years) of persistent cough with no cold (i.e. has months in which they cough on most days and cough on most days of as much as 3 months each year)

^c^Duration (measured in years) of persistent phlegm with no cold (i.e. has months in which they produce phlegm on most days and produce phlegm on most days of as much as 3 months each year)

^d^CAT scale: 0 (no issue) – 5 (severe and affects ability to function). Higher CAT score increases the likelihood of Chronic Obstructive Pulmonary Disorder

When participants with study-diagnosed OLD were asked to self-report chronic lung diseases, only 7 (20%) participants reported a previous physician diagnosed chronic lung disease; indicating 80% of participants were undiagnosed. Similarly, out of the 11 participants interviewed with study-diagnosed OLD, only three reported a physician diagnosis of OLD. Participants unaware of their lung health were made aware and referred to appropriate care providers. Additional to lung diseases, self-reported mental health conditions such as depression and anxiety were 35 and 29%, respectively (Table 4).

**Table 4:** Results from the baseline GAD-7, PHQ-8 and EQ-5D-3L questionnaires of the PROMPT Cohort participants

Questionnaire:	Participants with spirometry (*N* = 64)	All participants (*N* = 80)
**GAD-7**^b^		
No Anxiety^a^	48%	33.7%
Mild Anxiety	19%	30%
Moderate Anxiety	22%	18.8%
Severe anxiety	11%	13.8%
Missing data	0%	3.7%
**PHQ-8**^c^		
No significant symptoms^a^	25%	27.5%
Mild	39%	37.5%
Moderate	21%	20%
Moderately severe	13%	12.5%
Severe	2%	1.25%
**EQ-visual analogue scale**		
Mean	61.9	62.2
Range	3–100	3–100
Standard deviation	17.4	17

^a^Includes missing or refusal to answer

^b^GAD-7 - Generalized Anxiety Disorder (7-item Questionnaire with ≥10 score means a Probable Diagnosis of GAD)

^c^PHQ-8 - Personal Health Questionnaire (Depression Score)

Symptoms of moderate to severe anxiety (measured by GAD-7) were in 32.6% of the participants, whereas 33.8% had symptoms suggestive of moderate to severe depression (measured by PHQ-8) (Table 2). Mental health and stress appeared as a strong theme in the qualitative analysis. Participants with study diagnosed COPD often suggested they had high levels of stress due to strained access to basic resources (shelter, food, and income) and had experienced previous emotional, family, and physical trauma (Table 5).

**Table 5:** Self-reported comorbidities of the PROMPT cohort participants

Disease	All participants with spirometry (*N* = 64)	Participants without OLD (Fixed Ratio) on spirometry (*N* = 26)	Participants without OLD (LLN) on spirometry (*N* = 35)	Participants with OLD (Fixed Ratio) on spirometry (*N* = 38)	Participants with OLD (LLN) on spirometry (*N* = 29)	Participants with BD response on spirometry (*N* = 10)
Lung Disease^a^	21.88%	26.92%	22.86%	18.42%	20.69%	20%
Heart Disease	9.38%	11.54%	11.43%	7.89%	6.90%	0%
Hypertension	4.69%	3.85%	5.71%	5.26%	3.45%	0%
Diabetes	9.38%	11.53%	8.57%	7.89%	10.34%	20%
Stroke	4.69%	3.85%	2.86%	5.26%	6.90%	0%
Cancer	4.69%	3.85%	2.86%	5.26%	6.90%	0%
Schizophrenia	4.69%	3.85%	5.71%	5.26%	3.45%	0%
Depression	37.5%	42.31%	34.29%	34.21%	41.38%	30%
Anxiety Disorder	29.7%	30.77%	25.71%	28.95%	34.48%	10%
Bipolar Disorder	9.38%	11.54%	8.57%	7.89%	10.34%	0%
Seizures	6.25%	0%	0%	10.53%	13.79%	0%

^a^(Asthma, Cancer, Emphysema, COPD)

### Social determinants in people with obstructive lung disease

Overall two major themes emerged: 1) socioeconomic status and stress, and 2) social networks and related experiences of trauma. The level of income individuals had was often described as “just enough”. When further explored, majority relied on social assistance payments to support their housing (market rent) and turned to informal work (day jobs cleaning, handy work, and painting) and survival work (panhandling, sex work, and drug dealing) to afford food, and (public) transportation. For example, one participant described:

*“My [income] would be right in between … somewhat adequate … my rent is direct, but for my monthly bills, food, transportation it’s not [enough]. Some months, keeping up [with costs] is more of a struggle than others.”*

All individuals linked their socioeconomic situation to daily stress and reported using both negative coping strategies (substance use, isolation and avoidance) and positive coping strategies (exercise, connecting with social network and positive affirmations). The second major theme appeared through their extensive social networks and related traumatic experiences. Individuals’ social network consisted of family and friends, community organizations, and people with similar SES. Physical and emotional trauma experienced by participants were often connected to their family and friends (such as the death of loved ones, sexual abuse by trusted adults) or events involving law enforcement or hospital healthcare providers:

*“I’ve gone to the [hospital], told them about my wrist not feeling right, telling them about my elbow and arm, how badly twisted up it was by a cop. Then [they] still [did] not check [on it] or take x-rays or do anything. So, I’m kind of scared to go back to that healthcare service. If they’re not going to listen to me, then why should I go. Why should I listen to them?”*

Positive relationships with some family members and community health workers (nurses, workers) were also mentioned.

## Discussion

OLDs place a significant burden on patients and the healthcare system in Canada. On average, conditions such as COPD and asthma attribute to a large number of emergency room visits and hospital admissions [20], with the economic burden on the Ontario provincial healthcare systems amounting to $ 141 million CAN for asthma [21]. The burden of COPD exacerbations on Canada alone is estimated to be $646–736 million CAN/year [22]. These high costs have led to a growing effort to understand the prevalence and burden of OLD.

However, there is a lack of data on the urban poor population. In the PROMPT cohort, we observed a disproportionately high prevalence of spirometry diagnosed asthma (14–16%) and COPD (31–44%), regardless of spirometry interpretation strategy used. The prevalence seen in the PROMPT cohort is 2 and 3 times greater than the reported prevalence of asthma and COPD in the general Canadian population [20]. The prevalence of OLD in the PROMPT cohort is anywhere from 2 to 6 times greater than the prevalence found across cities in Australia, Norway, Germany, United Kingdom, USA and the Netherlands [23]. Similarly, the estimated prevalence of spirometry diagnosed OLD seen in the PROMPT cohort is approximately 2 to 3 times greater than the estimated prevalence seen in a 2004 study with participants experiencing homelessness [5] and a 2011 study with urban participants using drugs [24], both in the United States.

The observed differences in these studies and our results may potentially be attributed to the participant’s tobacco smoking status and severity. All participants in the PROMPT study were currently smoking cigarettes with the majority of participants having a history of greater than 20 pack-years (51%) and smoking for more than 21 years (57%). In both studies conducted in the US, their estimated prevalence’s were based on cohorts where 68 and 88% of participants self-reported as ‘current smokers’. It is possible the prevalence of OLDs in the PROMPT cohort is much greater than what is seen in the general and related populations due to the rate and severity of tobacco smoking.

Despite the common suggestion that smoking is the primary cause behind observed disparities [3, 4, 11], the stark difference continues to exist when comparing the urban poor population who smoke to the general Canadian population who smoke. When comparing the PROMPT cohort to the CanCOLD study [25, 26] (capturing the general Canadian population with COPD and who smoke), the urban poor represented by the PROMPT cohort were much worse off (see Additional File 1); including a significantly higher prevalence of spirometry-diagnosed COPD in the urban poor population who smoke (31–44%) than the general Canadian population who smoke (11–17%) [25, 26]. Poor quality spirometry may contribute to the high prevalence of OLD in this study, however efforts were made to improve spirometry quality, including six-day standardized training, with consistent quality performance evaluations [17]. More importantly, it is difficult to deny symptoms such as cough, phlegm, shortness of breath, and wheezing. Disease burden, including cough, phlegm and wheezing, was 2–3 times higher in the PROMPT group in comparison to the CanCOLD cohort that smokes (see Additional File 1). The mean CAT score, measuring COPD burden on everyday life, is almost triple in PROMPT participants (25.78) than in the CanCOLD participants (7.8), indicating PROMPT participants are highly symptomatic (total score ≥ 10 points) and experience greater disease burden [27]. In addition to OLD specific burden, majority of participants face comorbidities, such as mild to severe anxiety and depression, indicated by GAD-7 and PHQ-8 scores respectively.

The disproportionately high disease prevalence and co-morbidities were expected to contribute to the participant’s perception of their health and everyday experience. Yet in the semi-structured interviews, the disease specific burden and management of the disease was not central to the participant’s perceived health and day-to-day wellbeing. When asked about their health, participants with OLD focused on social determinants (such as income, trauma, social networks, education, Substance Use Disorder, mental health challenges, and food insecurity) rather than the management or symptoms related to their OLD. This is not surprising, as literature has shown SES to be a strong contributor to the severity and prevalence of OLD, rivaling the contribution of smoking [28–30]. However, these results differ from other qualitative studies looking at the lived experience of people with OLD, which highlight disease specific burden and management [31].

One reason that may explain why OLD specific burden was not highlighted in the post-study interviews was the alarming rates of undiagnosed OLD. Approximately 80% of participants in PROMPT that were diagnosed with an OLD (45–59%) reported no physician-diagnosed lung related disease, suggesting they are unaware of having an OLD. This is 4 times greater than the estimated rate of undiagnosed COPD and asthma in the general population in Canada [13]. Out of the 11 participants interviewed, 3 recognized OLD as a chronic disease they have. A similar study focusing on a US urban population that uses drugs found that 50% of their population were living with unrecognized OLD [24]. Often times, vulnerable groups have poor access to healthcare, are underdiagnosed and are undertreated, all translating to poorer health and poorer quality of life [32, 33]. However, much like SES and the other SDH, inequitable access to healthcare services are the result of structural and systematic inequities such as discrimination and stigma [34]. Together, these factors have serious implications on disease prevalence, burden, and overall quality of life.

Overall, these results suggest the presence of deep social inequities that create complex situations for people from the urban poor population living with an OLD. Addressing the SDH, in addition to standard treatments such as pharmacotherapy or physiotherapy, should be strongly considered by policy makers, researchers, and health care professionals when designing interventions to improve the quality of life of people in the urban poor population with OLDs. Currently, the Canadian Government’s action plan includes framework development and program funding [35]. While the framework recognizes the role of SDHs, the majority of their programing focuses on curbing tobacco smoking [36] and fails to recognize upstream factors we identified (such as food insecurity, trauma, housing issues and unreliable income) that dominate the day to day life of people with OLDs. One potential approach to address these components is Community-Based Participatory Action Research (CBPAR), as used in the PROMPT project [16, 37]. CBPAR engages participants as equal decision-makers and builds trust, producing inclusive research as recommended in the American Thoracic Society/National Heart, Lung, and Blood Institute Workshop Report [15]. Future studies are encouraged to explore the effectiveness of interventions that use these community-based approaches to combat social disparities evident in OLD prevalence.

### Limitations

A major limitation in this study is being a single center with a small sample size. Nonetheless, it is the first study demonstrating the OLD prevalence and disease burden measured by spirometry in the urban poor population. Furthermore, a selection bias could be present in recruitment as peer researchers utilized their social networks. For these reasons, our results should be generalized to populations in other settings across Canada with hesitancy. However, the demographic variables are similar to other cohort projects in the same population in North America, suggesting this cohort to be a representative sample of this population [38–41]. Additionally, our conscientious effort to utilize a social-network recruitment method in a community-based patient engagement approach helped to minimize mistrust, accessibility, and other common barriers faced by vulnerable populations when participating in research [7, 42].

The differences highlighted between the urban poor population and other populations (higher SES) may be considered to be obvious to some. However, this can lead to the growing exclusion of this population from potential research-based solutions for complex social and health related issues [43]. Lastly, we labelled participants with OLDs, including COPD and asthma only; the incidence of others, including bronchiectasis and chronic bronchitis, were not captured. Also, we did not capture the incidence of restrictive lung conditions, that may have an impact on the respiratory disease prevalence and burden in the urban poor population. Future studies are recommended to use respiratory, physiological, and radiological assessments, to help determine the complete picture of lung diseases in this underserved population.

## Conclusion

The most-vulnerable, low-income population of Ottawa, Canada is estimated to disproportionately experience a high prevalence and disease burden of OLD while facing challenging SDH. These findings suggest a significant health and social inequity, uncovering issues of poor social determinants of health and access to healthcare in Canada. Overall, this prompts the need for further research investigating the depth of these issues, and a new approach for clinical-based research, which integrate comprehensive, community-based programs for the most vulnerable populations.

## Supplementary Information

**Additional file 1.** General Population vs Urban Poor Population, PROMPT and CanCOLD: Shows comparison between PROMPT population and General Canadian Population, including discussion and 2 tables.

**Additional file 2.** Measures: Describes details on 3 measures used in study, including description, scoring and interpretation.

**Additional file 3.** Demographic Questionnaire: Co-designed with community peer researchers for the PROMPT project.

## Data Availability

Parent study (Management and Point-of-Care of Tobacco: A Community-Based Participatory Action Research Project, PROMPT) is available here (10.1136/bmjopen-2017-018416). The Clinical Trails registry for the PROMPT study can be found here (ClinicalTrails.gov Identifier: NCT03626064). All available data can be obtained by contacting the corresponding author.

## Abbreviations

COPD: Chronic obstructive lung disease
OLD: Obstructive lung disease
LLN: Lower limit than normal
PROMPT: Participatory Research in Ottawa: Management and Point-of-Care for Tobacco Dependence
SES: Socioeconomic status
SDH: Social determinants of health
