# Supplementary material for: Prevalence and burden of obstructive lung disease in the urban poor population of Ottawa, Canada: a community-based mixed-method, observational study
Source: BMC Public Health. 2021 Jan 21;21:183. doi: 10.1186/s12889-021-10209-w (PMC7819217; doi:10.1186/s12889-021-10209-w)
Supplement: Supplementary file 1 — Additional file 1. General Population vs Urban Poor Population, PROMPT and CanCOLD: Shows comparison between PROMPT population and General Canadian Population, including discussion and 2 tables. [file 12889_2021_10209_MOESM1_ESM.docx]

# **General Population vs Urban Poor Population: PROMPT and CanCOLD**

The demographic characteristics of the PROMPT sample were compared to similarly published data on the CanCOLD cohort. However, the anxiety, depression, and the quality of life scores between the cohorts were not relevant for comparison as they were measured differently: GAD-7 and PHQ-8 were used in PROMPT and HADS anxiety and depression scores were used in CanCOLD; EQ-5D-3L was used in PROMPT and the SF-36V2 in CanCOLD. The COPD prevalence was markedly higher at 44% among those who completed spirometry measurements in the PROMPT group (n=64) compared to 17% in the CanCOLD population, with 93% of the PROMPT participants experiencing moderate to severe OLD compared to 44% of the CanCOLD population (n=2598) (as determined by spirometer assessment and GOLD classification) (1–3). While asthma prevalence was the same (16%) between PROMPT population and the CanCOLD population it should be noted that the prevalence of asthma was determined by estimated prevalence from spirometry results showing significant post-bronchodilation reversibility and self-reported physician-diagnosis of asthma, respectively (4). Therefore, the ability to compare asthma prevalence between the two cohorts is limited.

An important issue surrounding the urban poor population that this study uncovers is the importance of representation in research. Though there are efforts to understand the burden of OLD, there is no comprehensive data on the urban poor. In Canada, the CanCOLD cohort includes participants through random digit dialing, to select representative samples of the general population (2). Therefore, both studies inherently exclude the urban poor as members of this population may be homeless or precariously housed with frequent changes to their address. They may also have limited access to a stable phone number and may face barriers to using a mobile phone, including their phone frequently getting lost, stolen or confiscated by the police force; common situations reported by the participants and peer researchers at *The Bridge*. Thus, the absence of an effort to include members of marginalized populations can result in systematic exclusion from research that can potentially improve health outcomes and reduce health inequities.

**References**

1. Labonte LE, Tan WC, Li PZ, Mancino P, Aaron SD, Benedetti A, et al. Undiagnosed chronic obstructive pulmonary disease contributes to the burden of health care use. Data from the CanCOLD study. Am J Respir Crit Care Med. 2016;194(3):285–98.

2. Buist AS, McBurnie MA, Vollmer WM, Gillespie S, Burney P, Mannino DM, et al. International variation in the prevalence of COPD (The BOLD Study): a population-based prevalence study. Lancet. 2007;370:741–50.

3. Tan WC, Bourbeau J, Hernandez P, Chapman KR, Cowie R, Fitzgerald JM, et al. Exacerbation-like respiratory symptoms in individuals without chronic obstructive pulmonary disease: results from a population-based study. Thorax. 2014;69(8):709–17.

4. Tan WC, Sin DD, Bourbeau J, Hernandez P, Chapman KR, Cowie R, et al. Characteristics of COPD in never-smokers and ever-smokers in the general population: results from the CanCOLD study. Thorax. 2015;thoraxjnl-2015.

Additional File 1, Table 1: Demographic Characteristics of Participants with Satisfactory Spirometric Measurements of the PROMPT and CanCOLD participants

| **Characteristic** | **PROMPT Participants**  **(n = 64)** | **PROMPT Ever-Smokers**  **(n =64)** | **CanCOLD**  **Participants**  **(n = 4893)***  **(Tan, 2015)** | **CanCOLD**  **Ever-Smokers**  **(n =2598)***  **(Tan, 2015)** |
| --- | --- | --- | --- | --- |
| **Sex (Male)** | 44 (69%) | 44 (70%) | 2096 (42.83%)* | 1205 (46.38%)* |
| **Ethnicity (Caucasian)** | 62 (78%) | 62 (78%) | 4475 (91.46%)* | 2456 (94.5%)* |
| **Age, mean** | 43.91 (-) | 43.91 (-) | 56.94£ (-) | 58.18* (-) |
| **Age**  **> 40**  **40-49**  **50-59**  **60-69**  **70+** | 15 (23.44%)  25 (39.06%)  21 (32.81%)  3 (4.69%)  0 | 15 (23.44%)  25 (39.06%)  21 (32.81%)  3 (4.69%)  0 | 0*  1374 (28.09%)*  1561 (31.90%)*  1175 (24.01%)*  783 (16.00%)* | 0*  672 (25.87%)*  811 (31.22%)*  674 (25.94%)*  411 (16.97%)* |
| **BMI, kg/m2, mean** | 25.96 (-) | 25.96 (-) | 27.89 (-)€ | 28.27 (-)* |
| **Pack years > 20** | 33 (51.56%) † | 33 (51.56%) † | 1231 (25.16%) | 1231 (47.38%)* |
| **Education, years** | - | - | 15.44 ‡ | 14.81 (-) |

4893/5176 participants in can COLD and 64/80 in PROMPT had spirometric measurements that satisfied ATS criteria. *CanCOLD References ^(4)^; ^£^ average age calculated between average age of never-smokers and ever-smokers. ^€^ average BMI calculated between average BMI’s of never-smokers and ever-smokers. ^†^ Pack years calculated from self-reported cigarettes/day and assuming 20 cigarettes/pack (# of packs/day x number of smoking years) ^‡^ average years of education calculated between average years of education between never-smokers and ever-smokers.

Additional File Table 2: Comparison of CanCOLD data and PROMPT data on Obstructive Lung Disease prevalence, disease burden and Quality of Life questionnaire scores.

| Variables | CanCOLD Data (N=2598*)  (Ever smokers) | PROMPT data (n=64^1^)  (Current smokers) | Confidence Interval at 95% for PROMPT cohort with spirometry (N=64) |
| --- | --- | --- | --- |
| COPD Prevalence  GOLD 1  GOLD 2+ | 17%^£^  56%^€^  44%^€^ | 44%  7%  93% | 33-55% |
| Asthma Prevalence | 16%* | 16% | 7-25% |
| Overall OLD Prevalence | 30%^€^ | 59% | 44-74% |
| Disease Burden |  |  |  |
| Cough | 22%^£^ | 64% | 51-77% |
| Phlegm | 19%^£^ | 70% | 58-82% |
| Shortness of breath | 38%^£^ | 39% | 27-51% |
| Wheezing | 45%^£^ | 71% | 59-83% |
| CAT Score | 7.8 ^€^ | 25.78 | - |

CanCOLD References: *(4); ^£^(2);^€^(3), ^1^COPD, asthma and overall OLD prevalence percentage is based on participants who completed spirometry measurements (N=64)
