# Supplementary material for: Prevalence and burden of obstructive lung disease in the urban poor population of Ottawa, Canada: a community-based mixed-method, observational study
Source: BMC Public Health. 2021 Jan 21;21:183. doi: 10.1186/s12889-021-10209-w (PMC7819217; doi:10.1186/s12889-021-10209-w)
Supplement: Supplementary file 2 — Additional file 2. Measures: Describes details on 3 measures used in study, including description, scoring and interpretation. [file 12889_2021_10209_MOESM2_ESM.docx]

# **Measures**

**Canadian Cohort Obstructive Lung Diseases (CanCOLD)**: The Burden of Lung Disease (BOLD) core questionnaire used in the CanCOLD study was administered to assess symptoms such as cough, phlegm, wheezing and breathlessness.

**COPD Assessment Test (CAT)**: The CAT is a validated simple 8-item disease-specific measure administered to assess the impact of COPD on health status. The CAT scoring ranges from 0-40.

**EQ-5D-3L:** The EuroQol questionnaire (EQ-5D-3L) is a frequently applied general health status measure. The EQ-5D-3L is a brief questionnaire used to evaluate the health-related quality of life for the study cohort. It consists of five dimensions: mobility, self-care, usual activities, pain/discomfort and anxiety/depression - which provides a single index value for health status for each individual. The participants were also given a visual analogue scale (EQ-VAS) and were asked to rate their health on a 20 cm vertical visual analogue scale ranging from 0-100, with 0 representing ‘the worst health you can imagine’ and 100 representing ‘the best health you can imagine’.
