# Supplementary material for: Prevalence and burden of obstructive lung disease in the urban poor population of Ottawa, Canada: a community-based mixed-method, observational study
Source: BMC Public Health. 2021 Jan 21;21:183. doi: 10.1186/s12889-021-10209-w (PMC7819217; doi:10.1186/s12889-021-10209-w)
Supplement: Supplementary file 3 — Additional file 3. Demographic Questionnaire: Co-designed with community peer researchers for the PROMPT project. [file 12889_2021_10209_MOESM3_ESM.docx]

**Participatory Research in Ottawa: Management and Point-of-care of Tobacco Dependence**

**The PROMPT Study**

| **PARTICIPANT ID:** |
| --- |

| **INTERVIEWER (initials):** |
| --- |

| **DATE: (d/m/y)** |
| --- |

| **INTERVIEW LOCATION:** |
| --- |

**PART I:**

**Demographics:**

In this first section, I will ask you a few questions so that we know a little bit more about you. Some of the questions ask about your ethnic background, age, and how you make money. We ask these questions because our goal is to provide better services for people who use drugs. Knowing this information will help us to do that because we will be able to figure out who we reached through our survey, whether there are similarities between groups, and how best to

organize community based health care services and supports.

**1. Do you identify as?**

- Male
- Female
- Trans male-to-female
- Trans female-to-male
- Two-spirited
- Other
- No answer

**2. What is your first language?**

- French
- English
- Other (Specify: ______________)
- No answer

**3. What was the highest level of education that you completed? (Check only one)**

- None
- Elementary/ grade school or some high school
- High school graduate/GED
- Some college or university
- College or university completed
- No answer

**4. Which ethnic group do you most identify yourself with?**

- Caucasian (white)
- Aboriginal (Indicate sub-group)
  - Métis
  - Inuit
  - First Nation (Specify: _______)
- Latino
- South Asian
- East Asian
- North African or Middle Eastern
- West, East or Sub-Saharan African
- Caribbean or Indo-Caribbean
- Black Canadian
- Other (Specify:___________)
- No answer

**5. If you identified as Aboriginal, have you or anyone in your family experienced the residential school system? (check all that apply)**

- Yourself
- Parent
- Grand-parent
- Aunt/Uncle
- Cousin
- Sibling
- Other (specify___________)
- No answer

**6. If you identified as Aboriginal, do you feel:**

- Mostly connected to an Aboriginal community
- Equally connected to an Aboriginal and non-Aboriginal community
- Not connected to community
- Mostly connected to a non-Aboriginal community
- No answer

**7. In which Country were you born?**

- _________________________
- If not Canada, what year did you move to Canada? _______________

**8. In which town or city were you born? __________________________**

- No answer

**9. How long have you been living in Ottawa?**

_____Years

_____ Months

____Weeks

- No answer

**10. What is your estimated monthly income (from all sources)?**

- Less than $499
- $500 to $999
- $1000 to $1999
- $2000 to $2999
- $3000 to $3999
- $4000 to $4999
- $5000 or more

**11. How much money to do usually spend on cigarettes every month?**

$__________

- No answer

**12. In the past 12 months, how often have you been hungry but unable to afford enough food?**

- Always (100% of the time)
- Most of the time (75-99%)
- Usually (50-75% of the time)
- Sometimes (25-50% of the time)
- Occasionally (<25% of the time)
- Never
- No answer

**PART II:**

**Smoking:**

**13. How long has it been since you last smoked tobacco?**

_________ (hours) _________(minutes) ________(days)

**14. What form of tobacco do you currently use? (Choose as many responses as applicable to you)**

- Cigarette
- Pipe
- Cigar
- Smokeless tobacco
- e-cigarettes

**15. Amount consumed daily/monthly: (Choose as many responses as applicable to you)**

**Daily Monthly**

_____ cigarettes _____ cigarettes

_____ cigars _____ cigars

_____ pipes _____ pipes

_____ chew _____ chew

_____ sniff _____ sniff

_____ e-cigarettes (no nicotine) _____ e-cigarettes (no nicotine)

_____ e-cigarettes (with nicotine) _____ e-cigarettes (with nicotine)

**16. Total number of years smoked _________ (years)**

**17. Where do you get your cigarettes from? (Choose as many responses as applicable to you)**

- Convenience store
- Local dealer

**18. Which type of cigarette do you smoke? (Choose as many responses as applicable to you)**

- Company Branded
- Native cigarettes

**19. How soon after you wake up do you smoke your first cigarette? ________ (minutes)**

**20. Do you find it difficult to refrain from smoking in places where it is forbidden (e.g., in church, at the library, in cinema, etc)?**

- Yes
- No

**21. Which cigarette would you hate most to give up? (Be sure to only select one response)**

- The first one in the morning
- Any other

**22. Do you smoke more during the first hours after waking than during the rest of the day?**

- Yes
- No

**23. Do you smoke even when you are ill enough to be in bed most of the day?**

- Yes
- No

**24. Do others smoke in the place you live or around you?**

- Yes
- No

**25. Do you wake up to smoke in the middle of the night?**

- Yes
- No

**26. Do you have to smoke if you wake up in the middle of the night for some other reason such as for using the washroom or for drinking water etc.?**

- Yes
- No

**27. How many quit attempts (lasting ≥ 24 hours) have you made in the past year?**

________ (attempts)

**28. On a scale of 1-5, how important is it to you to quit smoking/ remain smoke-free?**

**(5=most important)**

1 2 3 4 5

**29. On a scale of 1-5, how confident are you that you can quit smoking/ remain smoke-free?**

**(5=most confident)**

1 2 3 4 5

**30. Do you currently use any smoking cessation aids: (Choose as many responses as applicable to you)**

- Patch
- Inhaler
- Gum
- Lozenge
- Varenicline/Champix®
- Bupropion/Wellbutrin®

**31. Have you ever smoked something other than tobacco?**

- Yes
- No

*If* ***yes*, 29a. Please list them below with the approximate frequency of use:**

**List Ever Daily Monthly**

Crack / day / month

Weed / day / month

Heroin / day / month

Fentanyl / day / month

Oxycontine / day / month

_____________ / day / month

_____________ / day / month

**32. If you smoke crack, do you feel the need to re-cook it?**

- - Yes
  - No

*If* ***yes*, 30a. Why do you feel the need to re-cook the crack? (Choose as many responses as applicable to you)**

- - Different dealer/source____
  - Lack of quality____
  - To increase purity____
  - Other_________

**30b. If you re-cook crack, how often? (Be sure to only select one response)**

- - Always (100% of the time)
  - Most of the time (75-99%)
  - Usually (50-75% of the time)
  - Sometimes (25-50% of the time)
  - Occasionally (<25% of the time)
  - Never
  - No answer

**33. What type of device do you use to smoke crack? (Choose as many responses as applicable to you)**

- Homemade pipe___
- Stem____
- Ash pipe____

**34. What type of screen do you use to smoke the crack? (Choose as many responses as applicable to you)**

- - Ash__
  - Brilo__
  - Brass screen___
  - Stainless steel screen___

**35. How much money do you spend on crack?**

- $ ___ /day
- $ ___ /week
- $ ___ /month

**36. How soon after you wake up do you have to smoke crack?**

_________ minutes/hours

**37. Do you smoke crack at unsafe places?**

- - Yes
  - No

**38. Which crack puff would you hate most to give up?**

- The first one in the morning
- Any other

**INTERVIEWER’S COMMENTS:_______________________________________________**

**_______________________________________________________________________________________________________________________________________________________________________________________________________________________________________________________________________________________________________________________**

**INTERVIEWER’S INITIALS______________**
